# Deletion of rRNA Operons of *Sinorhizobium fredii* Strain NGR234 and Impact on Symbiosis With Legumes

**DOI:** 10.3389/fmicb.2019.00154

**Published:** 2019-02-13

**Authors:** Ala Eddine Cherni, Xavier Perret

**Affiliations:** Microbiology Unit, Department of Botany and Plant Biology, Sciences III, University of Geneva, Geneva, Switzerland

**Keywords:** nitrogen fixation, nodulation, competition, rhizosphere, colonization

## Abstract

During their lifecycle, from free-living soil bacteria to endosymbiotic nitrogen-fixing bacteroids of legumes, rhizobia must colonize, and cope with environments where nutrient concentrations and compositions vary greatly. Bacterial colonization of legume rhizospheres and of root surfaces is subject to a fierce competition for plant exudates. By contrast root nodules offer to rhizobia sheltered nutrient-rich environments within which the cells that successfully propagated via infection threads can rapidly multiply. To explore the effects on symbiosis of a slower rhizobia growth and metabolism, we deleted one or two copies of the three functional rRNA operons of the promiscuous *Sinorhizobium fredii* strain NGR234 and examined the impact of these mutations on free-living and symbiotic lifestyles. Strains with two functional rRNA operons (NGRΔrRNA1 and NGRΔrRNA3) grew almost as rapidly as NGR234, and NGRΔrRNA1 was as proficient as the parent strain on all of the five legume species tested. By contrast, the NGRΔrRNA1,3 double mutant, which carried a single rRNA operon and grew significantly slower than NGR234, had a reduced symbiotic proficiency on *Cajanus cajan, Macroptilium atropurpureum, Tephrosia vogelii*, and *Vigna unguiculata*. In addition, while NGRΔrRNA1 and NGR234 equally competed for nodulation of *V. unguiculata*, strain NGRΔrRNA1,3 was clearly outcompeted by wild-type. Surprisingly, on *Leucaena leucocephala*, NGRΔrRNA1,3 was the most proficient strain and competed equally NGR234 for nodule occupation. Together, these results indicate that for strains with otherwise identical repertoires of symbiotic genes, a faster growth on roots and/or inside plant tissues may contribute to secure access to nodules of some hosts. By contrast, other legumes such as *L. leucocephala* appear as less selective and capable of providing symbiotic environments susceptible to accommodate strains with a broader spectrum of competences.

## Introduction

Nitrogen-fixing symbioses between legumes and soil bacteria, commonly known as rhizobia, are responsible for introducing a large fraction of fixed N into terrestrial ecosystems. These beneficial plant-microbe associations come in many forms and shapes (Masson-Boivin et al., 2009; Sprent et al., 2013), yet all involve the intracellular colonization by soil rhizobia of legume cells grouped into nodules: Specialized root (or more rarely stem) organs within which endosymbiotic rhizobia reduce atmospheric nitrogen (N_2_). Although alternative mechanisms exist in diverse legume species, infection of root tissues is often mediated by infection threads (ITs) that guide dividing rhizobia through several cortical cell layers and toward the developing nodule primordia (Gage, 2004). In most legume crops, establishment of symbiosis is triggered and coordinated by molecular signals exchanged between legumes and rhizobia, beginning with the secretion of plant flavonoids to which compatible rhizobia respond with the synthesis and secretion of nodulation (Nod-) factors (NF). In turn, NF provoke the curling of root hairs onto which rhizobia are attached, the division of cortical cells that will eventually form nodule primordia, and permit the entry of rhizobia into root hairs (Perret et al., 2000; Oldroyd et al., 2011).

In *Medicago truncatula*, initiation of ITs begins once root hairs are fully curled and have entrapped, in most cases, a single rhizobia cell at the origin of an enclosed micro-colony. ITs begin to form 10–20 h after infection chambers are sealed (Fournier et al., 2015), and extend by 4–5 μm per hour with alternating phases of rapid or slower elongation depending on the intracellular dynamics of infected root hairs (Fournier et al., 2008). Successive cell divisions allow rhizobia to progress within developing ITs and microscopy studies showed that generation time of rhizobia within ITs was close to that observed *in vitro* using standard growth media (Gage, 2002). Thus, growth conditions within developing ITs must be favorable to rhizobia with enough nutrients to sustain a rapid bacterial division when needed. It is unknown whether some legumes, at one stage of the infection process, favor strains with either faster or slower generation times, however. In any event, bacterial surface components and secreted proteins play important roles in modulating the infection process (Jones et al., 2007). Once released from ITs, rhizobia multiply within the cytoplasm of nodule cells and eventually differentiate into N_2_-fixing bacteroids. Reduced nitrogen is assimilated by host plants in exchange for amino acids and carbon sources derived from photosynthesis that fuel the intense bacteroid metabolism (Poole et al., 2018).

Nodule morphology and characteristics vary considerably between legume species (Sprent et al., 2017). Major legume crops make either one of two nodule types that can be distinguished according to their ontogeny and development, however (Ferguson et al., 2010). Nodules of indeterminate growth possess a persistent distal meristem that, in *Medicago* species, derives from middle cortical cells with inner cortical and pericycle cells also contributing to nodule growth (Xiao et al., 2014). Mature indeterminate nodules (IDN) are elongated and characterized by a longitudinal gradient of plant and rhizobia cells at different stages of differentiation. Inside IDN of *M. truncatula* and other legumes of the Inverted Repeat-Lacking Clade (IRLC) (Montiel et al., 2017), nodule-specific cysteine-rich plant peptides provoke a profound and irreversible differentiation of bacteroids making them incapable of resuming free-living growth (Mergaert et al., 2006). Yet, inside IDN of the non-galegoid species *Mimosa pudica* (mimosoid clade of Caesalpinioideae), bacteroids were reported to resemble free-living cells in many aspects indicating that terminal bacteroid differentiation was not a general feature of IDN making legumes (Marchetti et al., 2011). By contrast, determinate nodules (DN) are spherical in shape, and reported to originate with external cortical cells and to have a transient meristematic activity, resulting in a differentiation of plant and rhizobia cells that is more synchronous throughout the nodule. As bacteroids of DN are not terminally differentiated, most rhizobia can resume a free-living growth in soils once nodules senesce and disaggregate. Recently, plants of the *Indigofera* and *Tephrosia* genera that were initially thought to form IDN, were reported to make nodules that carry secondary clusters of dividing cells instead of a persistent meristem (Ren, 2018). Yet, regardless of the strain or type of nodule considered, rhizobia must first efficiently colonize the plant rhizosphere, multiply within growing ITs, circumvent or withstand plant defenses, colonize and establish persistent colonies inside nodules cells, before becoming proficient symbionts. As a single rhizobia cell suffices to make a functional nodule containing up to 10^8^ to 10^9^ bacteroids (Kiers et al., 2003), numerous cell divisions and intense bacterial metabolism are needed to secure establishment of a proficient symbiosis.

Amongst the many rhizobia strains studied worldwide, *Sinorhizobium* (*Ensifer*) *fredii* strain NGR234 has the broadest host-range described so far (Pueppke and Broughton, 1999). Capable of nodulating plants of >120 legume genera, NGR234 fixes nitrogen inside nodules of determinate and indeterminate types. Such unsurpassed symbiotic promiscuity, raised the question of the molecular mechanisms used by NGR234 to elicit root nodule formation, successfully infect nodule cells and fix nitrogen on/in so many hosts (Broughton et al., 2000; Perret et al., 2003). Many studies confirmed that the 536 kb plasmid called pNGR234a carries most of the symbiotic genes [including (Broughton et al., 1986; Perret et al., 1991; Freiberg et al., 1997)], albeit the NGR234 3.9 Mb chromosome and 2.4 Mb megaplasmid pNGR234b were also shown to contribute to symbiosis (Viprey et al., 2000; Schmeisser et al., 2009). Strain NGR234 is not necessarily the most infective or proficient microsymbiont, however. For example, the slower growing *Bradyrhizobium japonicum* strain G49 outcompeted NGR234 on *Vigna unguiculata* (Ziegler et al., 2012), even though cowpea is considered a promiscuous legume (Lewin et al., 1987). Similarly, the slow-growing *B. japonicum* strain E109 also outcompeted the faster-growing *S. fredii* strain SMH12 on several soybean cultivars (Pastorino et al., 2015). Although many factors contribute to make strains more competitive than others for nodulation (Triplett and Sadowsky, 1992), a slower growth rate apparently does not reduce competitiveness of bradyrhizobia.

Fast-growing rhizobia (e.g., sinorhizobia, mesorhizobia, etc.) have generation times of 2–5 h, whereas slower growing strains such as bradyrhizobia may divide every 6–13 h (Keyser et al., 1982). Various genetic and environmental factors influence growth rates, yet bacteria with fewer ribosomal RNA (rRNA) operons tend to grow slower than those with many rRNA gene copies (Shrestha et al., 2007), with the number of rRNA operons ranging from 1 to 15 copies per genome (Klappenbach et al., 2001). In general, rRNA operons code for 5S, 16S, and 23S rRNA subunits and one or several tRNA genes. NGR234 has three such rRNA operons (Perret et al., 1991), and was shown to divide every 2.5 h on a minimal medium supplemented with succinate as a sole carbon source (Saroso et al., 1984). Some rhizobia have more than three rRNA operon copies, including the fast growing strains *Cupriavidus taiwanensis* LMG19424^T^ (two chromosomes and 5 rRNA operons) (Amadou et al., 2008), *Methylobacterium nodulans* ORS2060^T^ (one chromosome and 7 rRNA operons) (Marx et al., 2012) and *Paraburkholderia phymatum* strain STM815^T^ (two chromosomes and 6 rRNA operons) (Moulin et al., 2014), which take two to three days to form visible colonies on synthetic media. By contrast, a single rRNA operon containing strain such as *Bradyrhizobium diazoefficiens* USDA 110^T^ divides every 9.6 h (Keyser et al., 1982), while the extremely slow-growing *Bradyrhizobium liaoningense* isolates have generation times of up to 40 h (Xu et al., 1995). Studies on *Escherichia coli* showed that cells with deletions in one to two of the seven rRNA operon copies present in this species could grow at nearly maximal rate and remained viable even with as little as a single rRNA operon (Asai et al., 1999; Bollenbach et al., 2009). Yet, in competition experiments wild-type cells rapidly outgrew mutants deleted for one or two rRNA operons, indicating that the seven operons architecture was favored to secure a rapid adaptation to “feast and famine” conditions encountered by *E. coli* through its life-history (Stevenson and Schmidt, 2004). Experiments on *Bacillus subtilis* confirmed that mutants with only one out of ten rRNA operons grew poorly but nonetheless retained the ability to sporulate, albeit at lower frequencies than the wild type (Nanamiya et al., 2010). While these findings confirmed that bacteria remained viable even with a number of rRNA operons considerably reduced, complex cell processes (such as division and sporulation) were affected making rRNA-mutants less fit than parent strains.

Changes in nutrient availability have been shown to shape bacterial communities, with strains having fewer rRNA operons dominating older successional communities with scarcer resources (Nemergut et al., 2016), and with rRNA operon numbers becoming predictors of bacterial reproductive strategies (Roller et al., 2016). During their lifecycle, rhizobia must operate and compete in environments where resources and cell densities vary greatly, including in nutrient poor and thinly populated soils, rich but highly colonized root surfaces, to the particularly sheltered nutrient-abundant but highly specialized nodule cells. Given these markedly different environments within which rhizobia must operate, either as free-living or as endosymbiotic cells, we asked whether deletion of rRNA operons would significantly alter symbiotic proficiency of a promiscuous rhizobia strain. Accordingly, NGR234 was deleted of one and two of its three functional copies of rRNA operons and the effects of these deletions on growth, nodulation of several legume species, symbiotic nitrogen fixation and competition for nodulation were examined.

## Materials and Methods

### Microbiological Techniques

Plasmids and bacterial strains used in this study are described in Supplementary Table S1. NGR234 and derivative strains were grown at 27°C in/on rhizobia minimal medium supplemented with succinate (RMS) (Broughton et al., 1986) or tryptone yeast (TY) medium (Beringer, 1974). *E. coli* was grown at 37°C in/on LB. Antibiotics kanamycin, rifampicin, and spectinomycin were used at final concentrations of 50 μg ml^-1^. To compare growth of NGR234 and derivative mutants, 10^8^ cells from overnight pre-cultures at OD_600_ comprised between 0.3 and 0.5 were transferred to 50 ml fresh RMS cultures that were incubated for 3 days at 27°C and 190 rpm. For each strain, three replicate cultures were followed in parallel and OD_600_ was measured at regular intervals. Generation time was estimated during the exponential phase of growth, using as standard the NGR234 growth curve established by Broughton and Fellay (unpublished). Development of colonies formed by NGR234 and rRNA-deleted mutants was followed on TY and RMS plates using serial cell dilutions to obtain plates with ca. 10, 100, and 1000 colonies. After 5 and 7 days of incubation at 27°C for TY and RMS, respectively, the diameter of at least 30 isolated colonies per strain was measured using the Fiji image processing software (Schindelin et al., 2012).

### Sequence Verification and Deletion of the NGR234 rRNA Operons

**FIGURE 1:**
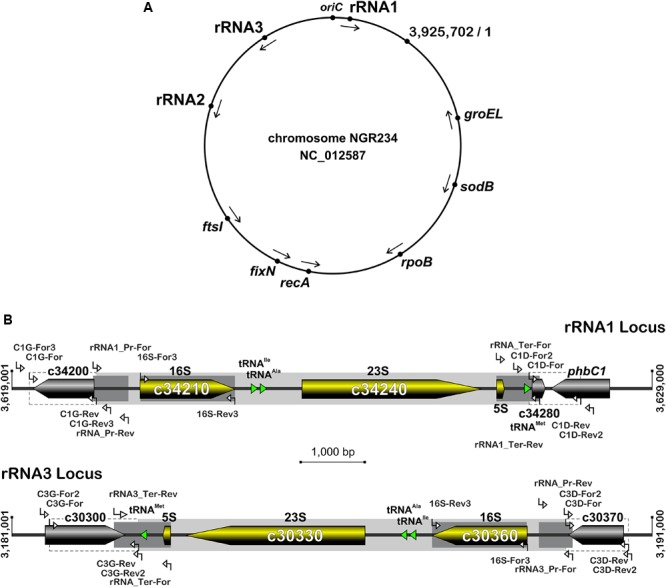
Organization of rRNA operons on NGR234 chromosome and detailed genetic maps of the rRNA1 and rRNA3 loci. **(A)** Circular map of the 3.9 Mb chromosome of NGR234 with the positions and orientations of the three rRNA operons as reported in the accession number NC_012587 (Schmeisser et al., 2009). **(B)** Detailed genetic maps of the rRNA1 and rRNA3 operons: Genes coding for 5S, 16S, and 23S rRNA subunits are shown as yellow arrows oriented according to transcription, while genes predicted to code for proteins are shaded in gray and those for tRNAs depicted as green triangles. The section of each operon that was replaced by Omega is shaded in gray. Segments of the 16S genes, promoters, and terminators that were verified by sequencing cosmid DNAs are highlighted in darker shades of gray. Regions flanking the deleted rRNA operons and used as sites for homologous recombination are delimited by dashed lines. Small and labeled white arrows represent the positions and orientations of primers used in this work.

To confirm sequence polymorphisms in promoter and terminator regions as well as in the 16S rRNA genes, overlapping clones of an ordered cosmid library (Perret et al., 1991) were used as templates to amplify each of the three rRNA operons independently: pXB123 and pXB487 were selected for amplifying the rRNA1 operon, pXB375 and pXB684 covered the rRNA2 locus, pXB72 and pXB942 carried the rRNA3 operon. End sequences of the inserts carried by these cosmids were obtained during the sequencing of the NGR234 genome (Schmeisser et al., 2009), allowing for a precise mapping onto the chromosome replicon (see Supplementary Table S1). Promoter and terminator regions were amplified and sequenced using combinations of primers listed in Supplementary Table S2 and which positions are shown in Figure 1. 16S rRNA genes were amplified and sequenced using the 16S-For3 and 16S-Rev3 primers (Fossou et al., 2016). To obtain the NGRΔrRNA3 mutant, regions flanking the rRNA3 operon were amplified using C3D-For/C3D-Rev and C3G-For/C3G-Rev primers (Supplementary Table S2) and NGR234 genomic DNA as template. The C3G-For/C3G-Rev and C3D-For/C3D-Rev amplicons were restricted by, respectively, *Spe*I and *Bam*HI or *Xho*I and *Bam*HI, and the resulting 1,391 and 979 bp fragments cloned into pBluescript KS+ (Stratagene) restricted by *Spe*I and *Xho*I, yielding construct pBSC3. The spectinomycin resistant (Sp^R^) Ω cassette of pHP45 (Prentki and Krisch, 1984) was cloned into the unique *Bam*HI site of pBSC3 to generate pBSC3Sp, which 4.4 kb *Spe*I-*Xho*I insert was further subcloned into pJQ200SK (Quandt and Hynes, 1993). The resulting pJQC3Sp clone was then mobilized into NGR234 by triparental mating using pRK2013 as helper (Figurski and Helinski, 1979). Eventually, mutants with the rRNA3 operon replaced by the Ω (Sp^R^) interposon were selected onto RMS plates containing spectinomycin (50 μg/ml) and 5% (w/v) sucrose. Candidate colonies for marker exchange were purified and genotyped by PCR using combinations of primers matching sequences outside of (C3D-Rev2 and C3G-For2) or within the deleted rRNA3 operon (C3D-For2 and C3G-Rev2), as well as within the Ω cassette (primer Omega) (see Supplementary Table S2 and Figure 1). A similar strategy was used to delete the rRNA1 operon and yield the NGRΔrRNA1 mutant, except that flanking sequences were amplified with the C1D-For/C1D-Rev and C1G-For/C1G-Rev primer pairs and were directly cloned into pJQ200SK using *Spe*I, *Bam*HI, and *Pst*I as restriction enzymes, yielding the pJQC1 intermediate construct (Supplementary Table S1). pJQC1Km was obtained by cloning the kanamycin resistant (Km^R^) Ω cassette into the unique *Bam*HI site of pJQC1. Following tri-parental mating and selection of candidate Km^R^ NGRΔrRNA1 mutants on sucrose-containing media, deletion of the rRNA1 operon was confirmed by PCR using combinations of the following primers: C1D-Rev2, C1G-For3, C1D-For2, C1G-Rev3, and Omega. Inserts cloned into pBSC3 and pJQC1 were verified by DNA sequencing. The mutant of NGR234 lacking both rRNA1 and rRNA3 operons (strain NGRΔrRNA1,3) was obtained by deleting the rRNA1 copy in the selected NGRΔrRNA3 mutant, using a similar procedure as described above. Genotype of NGRΔrRNA1,3 candidate mutants was verified by PCR, using primer sets designed to confirm deletion of the rRNA1 copy.

### Plant Assays

Nodulation assays were conducted as in Fumeaux et al. (2011), with plants of *Cajanus cajan, Leucaena leucocephala, Macroptilium atropurpureum* cv. Siratro, *Tephrosia vogelii*, and *V. unguiculata* cv. Red Caloona grown in Magenta jars containing vermiculite and watered using nitrogen-free B&D solution (Broughton and Dilworth, 1971). Seeds were surface-sterilized and incubated for 2–3 days in the dark, at 27°C and on B&D agar plates to germinate. Once germinated, seedlings were transferred to Magenta jars, two plants per pot, and each plantlet was inoculated 3 days later with a 200 μl water suspension of 2 × 10^8^ freshly grown rhizobia. Plants were grown in controlled conditions with a light phase of 12 h, a day temperature of 27°C, a night temperature of 20°C and in 60–70% humidity. At the end of each assay, shoots were harvested and dried at 60°C for 2 days prior to being weighted. Root systems were cleared of vermiculite and all nodules collected, counted and weighted, and then stored at -60°C until further use. Symbiotic proficiency of each strain was assessed by counting the total number of nodules, fresh weight of nodules and shoot dry weight of inoculated or control plants. To compare the kinetics of nodulation of NGR234 and rRNA-deletion mutants, *V. unguiculata* plants were grown as described above and harvested after 12, 24, and 36 days post-inoculation (dpi). At each time point, at least 12 plants were harvested for each treatment. In parallel, the relative chlorophyll content of first (between 12 and 21 dpi) and second (from 24 to 36 dpi) trifoliate leaves of *V. unguiculata* plants was monitored every third day using a Minolta SPAD-502 chlorophyll meter (Markwell and Blevins, 1999). For each inoculum, at least 8 plants in 4 pots were surveyed throughout the experiment with each time point corresponding to the mean of 48 measurements (2 readings per leaflet and 3 leaflets per plant). Trials to assess competition for nodule occupancy between inoculated strains were conducted as follows. Each *L. leucocephala* or *V. unguiculata* seedling was inoculated with a total of 10^6^ bacteria in a final volume of 200 μl. Appropriate amounts of single-strain inoculant were mixed to obtain final ratios of 1:1, 4:1, and 1:4 for NGR234 and NGRΔrRNA1, while NGR234 and NGRΔrRNA1,3 were only inoculated at 1:1 ratio. Respective titers of inoculated rhizobia were verified by plating aliquots of serial dilutions of each inoculum on TYA containing either rifampicin (all cells grew) or kanamycin (only mutants grew). To obtain nodules of sufficient size for subsequent analyses, plants of *L. leucocephala* and *V. unguiculata* were harvested at 28 and 24 dpi, respectively. Once harvested, root nodules were surface sterilized 3 min in 70% EtOH, washed three times in sterile H_2_O, 3 min in 4% NaClO, followed by six washes in sterile H_2_O. Surface sterilized nodules were then processed separately inside sterile microtiter plates with U-shape wells and thoroughly crushed in 70 μl of sterile distilled water. Nodule homogenates were replica-plated onto TY plates containing either rifampicin or kanamycin to identify nodules containing mutant strains. Growing colonies were examined after 6–8 days of growth at 27°C, using >140 nodules collected on more than 10 plants for each of the treatments.

## Results

### Structure and Organization of the NGR234 rRNA Operons

The rRNA operons flank both sides of the chromosomal replication origin with genes oriented as the replication forks (Figure 1A). All three rRNA operons have identical structures: One 16S rRNA gene at the 5′-end, followed by tRNA^Ile^ and tRNA^Ala^ copies, 23S and 5S rRNA genes and, at the 3′-end of the operon a copy of tRNA^Met^ (Figure 1B). According to published genome data (Schmeisser et al., 2009), polymorphisms in the three rRNA operons occur mostly in the promoter and terminator regions, with the rRNA3 differing most from the rRNA1 and rRNA2 copies. All three rRNA promoters include conserved -10 and -35 boxes that are separated by spacer sequences, of which only the rRNA3 copy differs by 7 out of 18 bp (see Supplementary Figure S1). Upstream of the conserved -35 hexamers, all three promoters include A-T rich sequences characteristic of upstream (UP) elements known to interact with the RNA polymerase complex and enhance promoter activity (Ross et al., 1993). NGR234 rRNA promoters include putative box A-like anti-termination and box B-like sequences (Berg et al., 1989), the latter of which form processing stalks with inverted repeats found downstream the 16S rRNA copies (Srivastava and Schlessinger, 1990). Terminator regions downstream of tRNA^Met^ sequences include conserved stem-loop structures preceded by series of adenines and followed by stretches of thymidine residues, characteristic of rho-independent type of transcriptional terminators (Farnham and Platt, 1981). Likewise its promoter region, the rRNA3 terminator region differs significantly from those of the rRNA1 and rRNA2 operons, with as many as 4 gaps and 79 polymorphic positions including in between the 5S rRNA and tRNA^Met^ genes and within the GC-rich palindromic terminator sequence (see Supplementary Figure S2). According to Schmeisser et al. (2009), all copies of 5S (NGR_c26480, NGR_c30320, and NGR_c34260) and 23S (NGR_c26490, NGR_c30330, and NGR_c34240) rRNA genes were identical whereas 16S rDNA sequences (NGR_c26520, NGR_c30306, and NGR_c34210) differed at eight nucleotide positions. Compared to consensus the 16S rRNA2 gene was reported to differ by one nucleotide while the 16S rRNA3 copy carried seven mismatches (see Supplementary Figure S3). Although sequence variations between 16S rRNA copies are not unheard of (Coenye and Vandamme, 2003; Acinas et al., 2004), allelic variations reported for NGR234 were verified by using as templates for 16S gene sequencing sets of overlapping cosmids that cover each of the three rRNA operons (Perret et al., 1991; Supplementary Table S1). Unexpectedly, sequencing data showed that copies of the 16S rRNA genes carried by the six cosmids were identical (Supplementary Figure S3). Thus, except for genuine differences in the promoter and terminator regions of copy 3, all of which were confirmed by sequencing cosmid templates, the rRNA operons of NGR234 are otherwise identical.

### Construction of rRNA-Deletion Mutants and Phenotypes on Free-Living Growth

**FIGURE 2:**
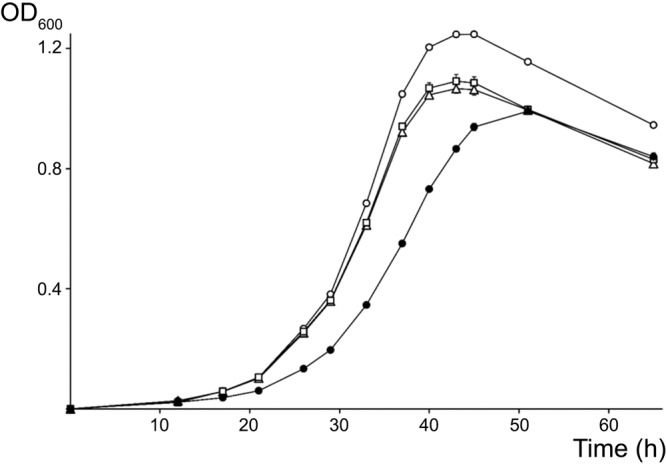
Growth curves for NGR234 and the rRNA-deletion mutants. Evolution of the growth of NGR234 (O), NGRΔrRNA1 (Δ), NGRΔrRNA3 (**□**), and NGRΔrRNA1,3 (●) strains in liquid RMS cultures at 27°C and with orbital shaking. Error bars indicate the standard deviation of the mean, with three independent cultures grown in parallel for each strain.

Because its promoter, 5S rRNA and tRNA^Met^ intergenic as well as terminator regions differed most from the other two copies, the rRNA3 operon was deemed as less likely to be essential for cellular processes and thus, was selected as the first target for mutagenesis. Accordingly, the complete rRNA3 operon was replaced by a spectinomycin-resistant version of the omega interposon (Prentki and Krisch, 1984). Given the NGRΔrRNA3 mutant was viable, the full rRNA1 operon was then selected as the second target for replacement by a kanamycin-resistant omega cassette. Eventually, after being deleted separately, the rRNA1 (Km^R^) and rRNA3 (Sp^R^) deletions were combined to yield the NGRΔrRNA1,3 mutant with a single rRNA2 operon. Development of parent and rRNA-deleted mutants was compared on/in rich TY and minimal RMS synthetic growth media. To observe the effect of deleting rRNA operons on the formation and development of isolated colonies on solid media, serial dilutions of exponentially growing cultures were plated onto TY and RMS agar (1.5% w/v) and incubated for 5 and 7 days, respectively. On TY agar, the diameter of NGR234 colonies was of 2.6 (±0.7) mm whereas NGRΔrRNA1 and NGRΔrRNA3 mutants formed colonies of 2.3 (±0.9) mm and 2.2 (±0.7) mm, respectively. By contrast, growth of NGRΔrRNA1,3 was significantly slower, with colonies reaching diameters of only 1.2 (±0.4) mm after 5 days of incubation (see Supplementary Figure S4). After 7 days on RMS agar, colonies of NGR234, NGRΔrRNA1, NGRΔrRNA3, and NGRΔrRNA1,3 had diameters of 2.6 (±0.5), 2.4 (±0.5), 2.6 (±0.6), and 1.5 (±0.3) mm, respectively. These results indicated that deletion of either of the rRNA1 or rRNA3 operons had a modest but noticeable impact on NGR234 viability and cellular growth on synthetic media whereas growth of NGRΔrRNA1,3 was considerably slower. Colony morphology remained unaltered, suggesting stability of mutated strains (Bollenbach et al., 2009). To better compare growth rates of the parent, NGRΔrRNA1, NGRΔrRNA3, and NGRΔrRNA1,3 mutant strains, cells were grown in RMS liquid cultures at 27°C and optical density of cultures was monitored during three consecutive days (see Figure 2). Mutant strains deleted for one rRNA operon grew at similar rates but slower than NGR234, while doubling time of NGRΔrRNA1,3 was, as expected, considerably extended. Based upon these measurements, generation time for each of the tested strains was estimated at 160 (±10) min for NGR234, 185 (±14) min for NGRΔrRNA3, 190 (±16) min for NGRΔrRNA1, and as much as 245 (±8 min) for NGRΔrRNA1,3.

### Effect of rRNA Deletion on Symbiosis With Legumes

To test the effect of deleting rRNA operons on nodulation and nitrogen fixation, the symbiotic proficiency of NGR234, NGRΔrRNA1, and NGRΔrRNA1,3 was examined on five legume species that formed nodules of either determinate (*M. atropurpureum* and *V. unguiculata*), indeterminate (*L. leucocephala*) or determinate with secondary clusters of dividing cells (*T. vogelii* and by analogy *C. cajan* as well) types. Phenotype of each inoculum was examined using nodule number (NN), total nodule fresh weight (NFW), and shoot dry weight (SDW) of inoculated plants as markers for symbiotic activity. As shown in Table 1, both of the NGRΔrRNA1 and NGRΔrRNA1,3 mutants still formed nodules (Nod+ phenotype) and reduced atmospheric nitrogen (Fix+ phenotype) on all of the hosts. SDW and NFW of plants inoculated with NGRΔrRNA1 or NGR234 were similar and only the NN of NGRΔrRNA1 showed a statistically significant decrease when inoculated onto *V. unguiculata*. By contrast, symbiotic proficiency of NGRΔrRNA1,3 was impaired on four out of the five legume species tested with NN, NFW, and/or SDW being significantly reduced (*P* < 0.05) when compared to plants inoculated with NGR234. Interestingly, when *L. leucocephala* plants were harvested at 49 dpi, NGRΔrRNA1,3 showed symbiotic efficacies similar to those of NGR234, NGRΔrRNA1, however.

**Table 1:** Symbiotic proficiency of NGR234, NGRΔrRNA1, and NGRΔrRNA1,3 on five legume species.

Host	Inoculum	Plants	mNN	mNFW (mg)	mSDW (mg)
*C. cajan* (42 dpi)	NGR234	12	25.1 (±6.7)	839.9 (±202.0)	1428.9 (±240.6)
	NGRΔrRNA1	12	24.3 (±6.6)	879.1 (±92.6)	1595.8 (±250.6)
	NGRΔrRNA1,3	12	20.4(±5.2)	658.7 (±182.6)*	1181.3 (±223.0)*
	Non-inoculated	8	0.0	0.0	100.5 (±22.2)
*M. atropurpureum* (42 dpi)	NGR234	10	60.6 (±12.6)	487.0 (±151.9)	783.6 (±182.3)
	NGRΔrRNA1	10	65.6 (±15.4)	498.8 (±87.9)	725.1 (±175.4)
	NGRΔrRNA1,3	12	49.3 (±11.2)*	431.7 (±159.1)	514.5 (±165.1)*
	Non-inoculated	6	0.0	0.0	42.8 (±16.5)
*L. leucocephala* (49 dpi)	NGR234	11	20.9 (±5.1)	168.0 (±38.4)	400.1 (±76.4)
	NGRΔrRNA1	11	20.4 (±5.1)	164.6 (±34.5)	399.2 (±89.8)
	NGRΔrRNA1,3	12	25.3 (±7.4)	148.4 (±28.8)	429.8 (±56.9)
	Non-inoculated	6	0.0	0.0	129.8 (±29.8)
*L. leucocephala* (70 dpi)	NGR234	12	40.4 (±11.1)	335.2 (±97.5)	647.6 (±136.1)
	NGRΔrRNA1,3	14	43.7 (±11.3)	489.3 (±150.2)*	1,001.1 (±290.5)*
	Non-inoculated	6	0.0	0.0	102.2 (±29.5)
*T. vogelii* (42 dpi)	NGR234	10	17.0 (±6.6)	596.0 (±171)	689.6 (±168.9)
	NGRΔrRNA1	11	16.0 (±5.5)	587.7 (±159.2)	683.0 (±175.5)
	NGRΔrRNA1,3	11	7.5 (±3.9)*	432.2 (±103.9)*	581.4 (±132.0)
	Non-inoculated	8	0.0	0.0	136.5 (±34.2)
*V. unguiculata* (36 dpi)	NGR234	10	111.0 (±26.8)	978.7 (±171.5)	1978.8 (±390.2)
	NGRΔrRNA1	11	89.4 (±13.5)*	893.4 (±224)	1730.4 (±369.7)
	NGRΔrRNA1,3	10	103.1 (±21.1)	646.7 (±99.5)*	1273.5 (±261.1)*
	Non-inoculated	4	0.0	0.0	193.3 (±79.4)

*Phenotype of each strain is reported as the mean nodule number (mNN), nodule fresh weight (mNFW), and shoot dry weight (mSDW) per plant, with standard deviation shown in parenthesis. Except for non-inoculated controls, results are the means of 10–12 plants per treatment, with each plant being inoculated with 200 μl of 2 × 10^*8*^ freshly grown bacteria. Depending on the host, plants were harvested at 36, 42, 49, or 70 days post-inoculation (dpi). Values that differed significantly between mutants and parent NGR234 (level α = 5%) are marked with an asterisk.*

### Kinetics of Nodulation of Parent and rRNA-Mutant Strains on *V. unguiculata*

**FIGURE 3:**
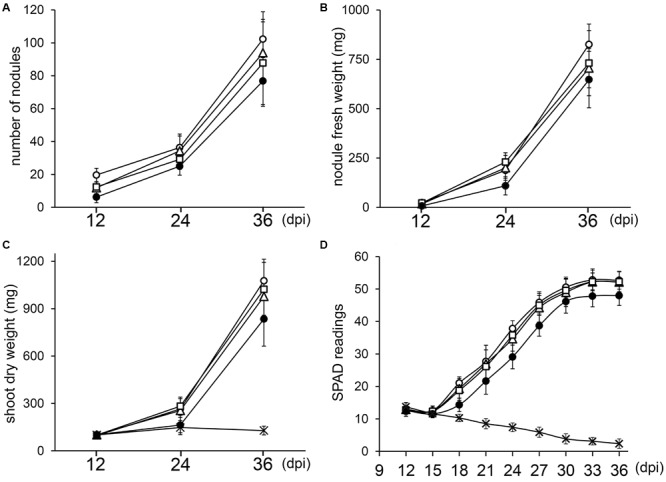
Kinetics of nodulation for NGR234 and rRNA-deletion mutants. Plants of *V. unguiculata* were harvested at 12, 24, and 36 days post-inoculation (dpi). For each time point, the nodule number **(A)**, nodule fresh weight **(B)**, and shoot dry weight **(C)** was averaged for 10–12 plants inoculated with either NGR234 (O), NGRΔrRNA1 (Δ), NGRΔrRNA3 (□), and NGRΔrRNA1,3 (●) strains. For non-inoculated control plants (X) that did not carry nodules, only the SDW and SPAD values are shown. Relative chlorophyll contents that are expressed as mean SPAD values for eight plants per treatment **(D)**, were measured every 3 days, starting at 12 dpi. Standard deviations are shown as bars above and below each data point.

Since deletion of one or two copies of the three rRNA operons of NGR234 did not block nodule formation or symbiotic nitrogen fixation, we examined whether kinetics of nodulation was altered. To this effect, *V. unguiculata* seedlings were inoculated with either NGR234, NGRΔrRNA1, NGRΔrRNA3, or NGRΔrRNA1,3 strains and parameters such as the average NN, NFW, and SDW for 12 plants per treatment were recorded at 12, 24, and 36 days post-inoculation (dpi). In addition, the relative chlorophyll content of the first and second trifoliate leaves was monitored every third day since 12 dpi, using a SPAD chlorophyll meter. At 12 dpi all inoculated plants already carried nodules (see Figure 3A and Supplementary Figure S5). Throughout the experiment, the NGRΔrRNA1 and NGRΔrRNA3 mutants formed slightly fewer nodules than NGR234, yet SDW of plants was similar indicating that symbiotic nitrogen fixation was not significantly impaired by deletion of a single rRNA operon (Figure 3C). Symbiotic proficiency of the NGRΔrRNA1 and NGRΔrRNA3 mutants at nearly wild type levels was also confirmed by SPAD readings, as relative chlorophyll contents of plants inoculated with NGR234 or mutants deleted of one rRNA operon were similar at all time points (Figure 3D). By contrast, at 12, 24, and 36 dpi the number of nodules formed by NGRΔrRNA1,3 (respectively, of 6.3, 25.0, and 76.8 nodules) was considerably reduced as compared to NGR234 (19.7, 36.4, and 102.3 nodules), suggesting nodule formation was significantly delayed (Figure 3A). Overall symbiotic activity of NGRΔrRNA1,3 was clearly impaired as shown in Figure 3C,D, with SPAD readings of inoculated plants that plateaued at 47.9 at 33 dpi whereas those inoculated with NGR234, NGRΔrRNA1, or NGRΔrRNA3 mutants reached higher values comprised between 52.2 and 52.8 (Figure 3D). To check for leghemoglobin expression, several nodules formed at 12, 24, and 36 dpi were sectioned and photographed. At 12 dpi, nodules formed by NGR234, NGRΔrRNA1, and NGRΔrRNA3 already contained leghemoglobin whereas most of the fewer nodules formed by NGRΔrRNA1,3 were still white or faintly pink (see Supplementary Figure S5). At 24 dpi, all of the nodules elicited by NGRΔrRNA1,3 had turned pink, indicating nitrogen fixation had started as confirmed by the increased SPAD values. At 36 dpi and regardless of the inoculum, nodules formed by all mutant or parent strains expressed leghemoglobins.

### Competition for Nodule Occupancy by NGR234 and rRNA-Deleted Strains

To determine whether deletion of rRNA operons influenced the competitiveness of NGR234 to form and occupy nodules, seedlings of *V. unguiculata* were inoculated with 10^6^ cells of NGR234 and NGRΔrRNA1 at respective ratios of ca. 1:1, 1:4, and 4:1. At 24 dpi, all nodules found on roots of inoculated plants were collected and bacteria isolated from >180 nodules per treatment. Isolated nodule bacteria were then tested for their resistance to kanamycin and/or rifampicin, to differentiate between the NGRΔrRNA1 (Km^R^ and Rif^R^) and NGR234 (Rif^R^) strains. Proportion of wild type versus mutant in each of the inocula was verified at the time of inoculating plants, showing that number of NGRΔrRNA1 cells was always slightly in excess when compared to NGR234 (see Table 2). Yet, regardless of the cell ratios that were inoculated, the most abundant strain always colonized nodules more efficiently, suggesting that deletion of the rRNA1 copy had no significant effect on the ability of NGR234 to nodulate *V. unguiculata*. However, when a similar experiment was conducted using NGR234 and NGRΔrRNA1,3 as competitors, the parent strain clearly outcompeted NGRΔrRNA1,3 on cowpea. Given the similar symbiotic proficiencies of NGR234 and rRNA-deletion mutants observed 49 dpi on *L. leucocephala* (Table 1), seedlings were inoculated with NGR234, NGRΔrRNA1,3 or a 1:1 mixed inoculant of NGR234 and NGRΔrRNA1,3. At 28 dpi, *L. leucocephala* plants challenged with the mixed inoculum were harvested and identity of nodule bacteria determined. As shown in Table 2, NGRΔrRNA1,3 occupied nearly half of the nodules collected at 28 dpi indicating the mutant was apparently as competitive as the wild type for infecting *L. leucocephala* nodules. Interestingly, when plants treated with single-strain inoculants were harvested at 70 dpi, the dry mass of *L. leucocephala* shoots inoculated with NGRΔrRNA1,3 was in average significantly higher than when NGR234 was used as inoculum (Table 1). These results confirmed that the effect on symbiosis of deleting rRNA operons of NGR234 varied considerably between host plants.

**Table 2:** Competition of NGR234 and rRNA-deletion mutants for nodulation of *V. unguiculata* and *L. leucocephala*.

	Inoculated cell ratios			Growing nodule isolates		
Competing strains	Aimed	Real	Host plant	Total	Km^R^	% Km^R^
NGR234 vs. NGRΔrRNA1	1:1	42:58		187	133	71.1
	4:1	70:30	*V. unguiculata*	241	54	22.4
	1:4	14:86		235	208	88.5

NGR234 vs. NGRΔrRNA1,3	1:1	43:57	*V. unguiculata*	147	5	3.4
	1:1	45:55	*L. leucocephala*	140	69	49.3

*To compare NGR234, NGRΔrRNA1, and NGRΔrRNA1,3 nodulation competitiveness, plants were challenged with mixed inoculants. On V. unguiculata, NGR234 and NGRΔrRNA1 were inoculated at ratios of 1:1, 1:4, and 4:1 for a total of 10^6^ cells per plant. Given its slower growth and poor competitiveness in initial tests on cowpea, the NGRΔrRNA1,3 mutant was inoculated at equal numbers with NGR234. For each inoculum, strain ratio was verified by plating serial dilutions of the inoculum onto TYA plates containing rifampicin or kanamycin, with kanamycin resistant (Km^*R*^) colonies or nodule isolates scored as being or containing mutant strains. For these analyses, nodules were collected on a minimum of 10 plants per treatment and at 24 and 28 dpi for V. unguiculata and L. leucocephala, respectively.*

## Discussion

Previous studies have addressed the impact of modifying the number or sequence of rRNA operons on various cell processes such as response of *Synechococcus* cyanobacteria to temperature changes (Monshupanee et al., 2006), resistance to clarithromycin and spectinomycin in *Mycobacterium smegmatis* (Sander et al., 1996), relative fitness and competition in *E. coli* batch and chemostat cultures (Stevenson and Schmidt, 2004), *E. coli* cell morphology (Asai et al., 1999) or sporulation of *B. subtilis* (Yano et al., 2013). To our knowledge, this is the first attempt at testing the effect of deleting rRNA operons on the growth and symbiotic behavior of rhizobia. NGR234 carries three functional rRNA operons that, unlike previously reported in Schmeisser et al. (2009), include identical copies of 16S rRNA genes (NGR_c26520, NGR_c30306, and NGR_c34210). Genuine polymorphisms between NGR234 rRNA operons were confirmed for the promoter and terminator regions, with the rRNA3 copy differing most from consensus, however. According to published genome sequences deposited in GenBank, intragenomic heterogeneity in rRNA operons also exists in several *S. fredii* strains including HH103 (NC_016812), USDA257 (NC_018000), and CCBAU 25509 (GCA_000264885 assembly). In fact, amongst the 7 *S. fredii* genomes archived in GenBank as complete replicons (strains CCBAU 25509, CCBAU 45436, CCBAU 83666, NGR234, and NXT3) or as high quality permanent drafts (strains HH103 and USDA 257), only CCBAU 25509 was found to carry a 16S rRNA gene that differed by one mismatch from the other two copies. By contrast variations between allelic 5S rRNA and tRNA^Met^ intergenic sequences appear to be more frequent, since *S. fredii* strains CCBAU 25509 and NXT3 (NZ_CP024307) also include polymorphic positions in one of the three corresponding regions of these genomes. With 4 deletions of up to 59 nucleotides and 79 additional mismatches that were all confirmed by cosmid sequencing, the 5S and tRNA^Met^ intergenic region of the NGR234 rRNA3 operon is strikingly divergent from the other two copies (see Supplementary Figure S2). In fact, probing of the Rhizobiaceae sequences archived in GenBank (in October 2018) showed the 448 bp long sequence between the 5′-end of 5S rRNA3 (NGR_c30320) to the 3′-end of the tRNA^Met4^ (NGR_c30310) better matched sequences of *Ensifer adhaerens* strains OV14 (CP007236) and Casida A (on plasmid pCasidaAB; CP015882) than those of the rRNA1 and rRNA2 operons of NGR234. Whether this 5S and tRNA^Met^ intergenic region of the NGR234 rRNA3 operon is an example of a convergent sequence evolution in distinct species of plant-interacting bacteria or results from a fusion of chromosome segments belonging to distinct strains is unknown, however.

To minimize any detrimental effect of altered stoichiometry in 5S, 16S, and 23S rRNA molecules on the assembly and functioning of ribosomes, mutants of NGR234 were deleted for whole rRNA1 and/or rRNA3 operons including for the corresponding promoter and terminator regions. As the Omega interposon is flanked by strong transcription terminators (Fellay et al., 1987; Fumeaux et al., 2011), polar effects on genes/operons immediately upstream and downstream of the inserted Omega cassette(s) are unlikely. Removal of entire rRNA operons also resulted in deleting copies of the tRNA^Ile^, tRNA^Ala^, and tRNA^fMet^ genes embedded within these transcription units, however. The genome of NGR234 was initially predicted to code for a set of 53 tRNAs, including a tRNA^Met^ copy (NGR_a03100) carried by the symbiotic plasmid pNGR234a (Schmeisser et al., 2009). NGR_a03100 was predicted in a region of pNGR234a that was found to be transcribed in free-living and endosymbiotic bacteria (Bakkou, 2011), and to be conserved in the symbiotic plasmids of strains *S. fredii* CCBAU 25509 (pSF25509a), CCBAU 45436 (pSF45436a), CCBAU 83666 (pSF83666a), HH103 (pSfHH103d), and USDA257 (pUSDA257) as well as in strains *Ensifer sojae* CCBAU 05684 (pSJ05684a) and *Sinorhizobium* sp. CCBAU 05631 (pSS05631a). GtRNAdb currently lists a total of 54 tRNAs for NGR234, with an additional tRNA^Ser^ (for TCG codon) on the minus strand of positions 3,328,552–3,328,463 of the chromosome and in place of the NGR_c31650 protein-coding gene, however (Chan and Lowe, 2016). According to GtRNAdb, the NGR234 chromosome codes for a single tRNA^Met^ (NGR_c27530) whereas NGR_c22590 and NGR_a03100 products, which were initially also annotated as tRNA^Met^, are now predicted to be modified by a tRNA^Ile^-lysidine synthetase and converted into AUA codon-specific tRNAs for isoleucine (Soma et al., 2003). Thus, derivative strains of NGR234 deleted for rRNA operons will also have fewer isoleucine and alanine tRNAs for AUC and GCA codons, respectively.

Nonetheless mutants of NGR234 carrying two or only one rRNA operons were viable. Deletion of a single rRNA operon had little effect on the growth of mutants, with generation times of 25–30 min longer than for the parent NGR234 strain (ca. 2h40). This suggests NGRΔrRNA1 and NGRΔrRNA3 possibly compensated for deletion of one rRNA operon by an increased expression of the remaining intact copies as was reported to occur in *E. coli* (Condon et al., 1993). Slower growth of NGRΔrRNA1 and NGRΔrRNA3 was also noticeable on solid media, suggesting both mutants may eventually colonize root systems of host plants less rapidly than parent strain. With only a single rRNA operon left, NGRΔrRNA1,3 showed a more pronounced growth defect, however. Such a difference in growth between the NGRΔrRNA1 and NGRΔrRNA1,3 mutants confirmed that, in spite of the intragenomic heterogeneity of promoter and terminator sequences discussed above, the rRNA3 operon is expressed and contributes substantially to cell metabolism. In the cyanobacterium *Synechococcus* strain PCC 7942, which chromosome encodes two rRNA operons (called *rrnA* and *rrnB*), deletion analyses suggested *rrnA* synthesized more rRNA and tRNA molecules than *rrnB* (Monshupanee et al., 2006). Whether all rRNA operons of NGR234 contribute equally to rRNA and tRNA pools is unknown, but our results confirmed all three copies are active and contribute to cell growth. Thus, a reduction in the number of rRNA operons resulted in a concomitant slower growth of NGR234, regardless of whether bacteria were in/on rich or poor medium. This observation is consistent with previous reports of slower growth following multiple deletions of rRNA operons in *E. coli, B. subtilis*, or *Synechococcus* (Asai et al., 1999; Monshupanee et al., 2006; Yano et al., 2013; Gyorfy et al., 2015).

Deletion of a single rRNA operon had little impact on the symbiotic proficiency of NGR234 on five different legume species, with NGRΔrRNA1 forming as many nodules as the parent strain. Nitrogen fixation was not impaired either since shoot dry weight of plants inoculated with NGR234 or NGRΔrRNA1 was similar and SPAD values showed *V. unguiculata* plants did not suffer from nitrogen starvation once nodules were formed and fully active, and as soon as 18 dpi. By contrast, deletion of both rRNA1 and rRNA3 operons significantly impaired but did not abolish proficiency of NGRΔrRNA1,3 on several hosts. The symbiotic defect of NGRΔrRNA1,3 was more pronounced on *M. atropurpureum* and *V. unguiculata* with a >30% decrease in SDW, whereas *C. cajan* and *T. vogelii* plants only suffered a ca. 15% decrease in SDW. Surprisingly, when plants were harvested 10 weeks post inoculation NGRΔrRNA1,3 was found to be more proficient than NGR234 on *L. leucocephala*, thus confirming that the impact on symbiosis of deleting rRNA operons is host-specific. *M. atropurpureum* and *V. unguiculata* form DN within which the intracellular rhizobia undergo multiple rounds of division inside plant cells that are themselves dividing. Once cell divisions eventually stop, mature nodules with fully differentiated bacteroids are formed (Ferguson et al., 2010). By contrast, IDN of *L. leucocephala* possess a persistent apical meristem and bacteria that are released from infection threads into plant cells that have stopped dividing will only divide a few times before differentiating into nitrogen-fixing bacteroids (Oke and Long, 1999). Recently, IDN of *Indigofera* and *Tephrosia* species were reclassified as DN that develop from the outermost root cortical cells and have, instead of persistent meristems, secondary clusters of dividing nodule cells some of which contain infection threads (Ren, 2018). Whether the differences in the infection process and ontogeny of determinate versus indeterminate nodules may explain the distinct symbiotic phenotypes of NGRΔrRNA1,3 on *V. unguiculata* and *L. leucocephala* remains to be explored.

Differentiation of rhizobia into bacteroids was reported to correlate with a reduction in translation machinery and an overall slowdown of bacteroid metabolism (Barnett et al., 2004; Becker et al., 2004). These studies were carried out on *S. meliloti* strain 1021, a symbiont of alfalfa that undergoes a strong endoreduplication process once released into nodule cells (Mergaert et al., 2006). Yet, a slower metabolism focused on symbiotic nitrogen fixation is most probably a feature characteristic of bacteroids in many legume hosts, in addition to auxotrophy for several amino acids and dependence for homocitrate and dicarboxylic acids provided by plants (Udvardi and Poole, 2013). Once rhizobia are established within nodule cells and have ceased to divide, transcription and translation seem to primarily target functions required for nitrogen fixation (Capela et al., 2006; Karunakaran et al., 2009), hence transforming bacteroids into nitrogen-fixing organelle-like entities. That NGR234, NGRΔrRNA1 and NGRΔrRNA3 share similar symbiotic phenotypes suggests mutants carrying two out of three rRNA operons can successfully meet the requirements for nitrogen fixation inside many hosts. With the loss of two out of three rRNA operons, nodulation became impaired on several legume species as shown by the significantly lower number of nodules formed by NGRΔrRNA1,3 on *C. cajan, M. atropurpureum*, and *T. vogelii* (Table 1) and the leghemoglobin-free nodules observed at 12 dpi on roots of *V. unguiculata*. Nonetheless, SPAD measurements showed that *V. unguiculata* plants inoculated with NGRΔrRNA1,3 already benefited from symbiosis as early as 18 dpi (Figure 3D). This beneficial input of NGRΔrRNA1,3 bacteroids was insufficient to translate into a stronger shoot growth, as SDW of inoculated and non-inoculated control plants remained similar at 24 dpi, however (Figure 3C). At 30 dpi and later, SPAD values measured for plants inoculated with NGRΔrRNA1,3 plateaued toward the maximum of ca. 48 units, and never reached the higher values of up to 52 units that were measured for plants inoculated with NGR234, NGRΔrRNA1 and NGRΔrRNA3. Thus, several factors seem to contribute to the lower proficiency of NGRΔrRNA1,3 on cowpea: a delay in nodule formation and in the establishment of fixing nodules, as well as a reduced capacity of the NGRΔrRNA1,3-infected nodules to sustain plant development over a longer period. Yet, the NGRΔrRNA1,3 and *L. leucocephala* association appeared as fully functional (Table 1), indicating the minimal requirements for symbiosis and dynamics of nodulation and nitrogen fixation must differ considerably between legume species. Whether *L. leucocephala* uses yet unknown compensation mechanisms to suppress the NGRΔrRNA1,3 deficiencies, allows a greater colonization of nodule cells by the mutant or is globally a less-demanding host for rhizobia capable of nodulation remains to be elucidated.

Many factors contribute to make rhizobia competitive for nodulation. For examples production of rhizobitoxine by *Bradyrhizobium elkanii* USDA94 strain enhances its competitive nodulation on *M. atropurpureum* (Okazaki et al., 2003). Utilization of several carbon sources has been shown to be important for competing for nodule occupancy in *R. leguminosarum* and *S. meliloti* (Fry et al., 2001; Kohler et al., 2010; Ding et al., 2012); Cell surface components such as exo- or lipo-polysaccharides contribute to *S. meliloti* ability to compete for nodule occupancy (Lagares et al., 1992; Geddes et al., 2014); General stress response (GSR) resulting in the induction of protection or damage repair functions in *B. diazoefficiens* 110^T^ are crucial for synchronization of infection thread formation, colonization, and nodule development (Ledermann et al., 2018); and presence of non-symbiotic bacteria such as *Acinetobacter* spp., *Enterobacter aerogenes, Klebsiella pneumoniae*, and *Pseudomonas marginalis* reduced alfalfa nodulation by *S. meliloti* (Li and Alexander, 1986). Deletion of a single rRNA operon, which slightly slowed growth and made mutants enter stationary phase earlier (see Figure 2) did not impair NGRΔrRNA1 competitiveness for nodulation on *V. unguiculata*. In fact, NGRΔrRNA1 occupied more nodules than NGR234 (71% versus 29%) when both strains were co-inoculated at roughly similar titers. Furthermore, while the slower dividing NGRΔrRNA1,3 occupied only 4% of *V. unguiculata* nodules, it successfully infected half of *L. leucocephala* nodules when co-inoculated with the parent at nearly 1:1 ratio. Thus, slower growth is not necessarily detrimental to competition for nodulation, as shown in a different context by the ability of a slow-growing *Bradyrhizobium* strain E109 to outcompete the fast-growing *E. fredii* strain SMH12 on soybean (Pastorino et al., 2015). Given that NGR234 and derivative mutants share otherwise identical repertoires of housekeeping and symbiotic genes, we expected the deletion of two out of three rRNA operons to considerably affect the fitness of NGRΔrRNA1,3 with possibly pleiotropic effects on cell division, metabolism and motility, and as a consequence on symbiotic proficiency. Yet, NGRΔrRNA1,3 performed surprisingly well as a single inoculum on all of the five legume species tested so far, and in particular on *L. leucocephala* where it showed improved proficiency when compared to NGR234. Together these results highlight the apparent robustness of symbiotic nitrogen fixation, but also how certain legume hosts can accommodate bacteroid deficiencies, as was recently illustrated by the unusual phenotype of the NGRΔ*nifQ* mutant on several species (Saad et al., 2018).

## Author Contributions

XP and AEC conceived the experiments, analyzed the data, and wrote the manuscript. AEC performed the experiments. Both authors have read and approved the final manuscript.

## Conflict of Interest Statement

The authors declare that the research was conducted in the absence of any commercial or financial relationships that could be construed as a potential conflict of interest.
