# Supplementary material for: Deletion of rRNA Operons of Sinorhizobium fredii Strain NGR234 and Impact on Symbiosis With Legumes
Source: Front Microbiol. 2019 Feb 13;10:154. doi: 10.3389/fmicb.2019.00154 (PMC6381291; doi:10.3389/fmicb.2019.00154)
Supplement: Supplementary file 1 [file Data_Sheet_1.PDF]

Supplementary Material to

Deletion of rRNA operons of *Sinorhizobium fredii* strain NGR234 and impact on symbiosis with legumes

By **Ala Eddine Cherni** and **Xavier Perret***

*** Correspondence:** Corresponding Author: xavier.perret@unige.ch

Table S1. List of bacterial strains and plasmids used in this study.

Strain, plasmid	Relevant properties	Source or reference
**Strains**		
NGR234	Rif^R-derivative of the strain isolated by M. J. Trinick	Stanley et al. 1988
NGRΔrRNA1	mutant of NGR234 in which chromosome positions 3'620'316 to 3'627'080 were replaced with Omega, Rif^R, Km^R	This work
NGRΔrRNA3	mutant of NGR234 in which chromosome pos. 3'182'978 to 3'189'650 were replaced with Omega, Rif^R, Sp^R	This work
NGRΔrRNA1,3	mutant derived from NGRΔrRNA3 in which the rRNA1 operon was also deleted, Rif^R, Km^R, Sp^R	This work
**Plasmids**		
pBluescript KS	ColEI-based phagemid, lacαZ^+, Ap^R	Stratagene
pJQ200SK	Versatile suicide vector, Gm^R	Quandt and Hynes, 1993
pRK2013	Tra^+ helper plasmid, Km^R	Figurski and Helinski, 1979
pHP45	Vector carrying either of the Ω Km^R or Ω Sp^R interposons	Prentki and Krisch, 1984
pBSC3	pBluescript clone carrying the 979 bp *Xho*I-*Bam*HI and 1,391 bp *Bam*HI-*Spe*I fragments that border the rRNA3 operon	This work
pBSC3Sp	pBSC3 derivative in which the Ω Sp^R interposon was inserted into the *Bam*HI site	This work
pJQC1	pJQ200SK clone carrying the 1,040 bp *Spe*I-*Bam*HI and 802 bp *Bam*HI-*Pst*I fragments that border the rRNA1 operon	This work
pJQC1Km	pJQC1 derivative in which the Ω Km^R interposon was cloned into the *Bam*HI site	This work
pJQC3Sp	4.4 kb *Spe*I-*Xho*I insert of pBSC3Sp cloned into pJQ200SK	This work
pXB72	Lorist2 cosmid covering positions 3,162,701 to 3,198,050 of the NGR234 chromosome with the rRNA3 operon, Km^R	Perret et al. 1991
pXB123	Lorist2 cosmid covering pos. 3’605’914 to 3’639’646 of the NGR234 chromosome with the rRNA1 operon, Km^R	Perret et al. 1991
pXB375	Lorist2 cosmid covering pos. 2’755’318 to 2’795’742 of the NGR234 chromosome with most of the rRNA2 operon, Km^R	Perret et al. 1991
pXB487	Lorist2 cosmid covering pos. 3’590’370 to 3’629’983 of the NGR234 chromosome with the rRNA1 operon, Km^R	Perret et al. 1991
pXB684	Lorist2 cosmid covering pos. 2’727’454 to 2’763’009 of the NGR234 chromosome with the rRNA2 operon, Km^R	Perret et al. 1991
pXB942	Lorist2 cosmid covering pos. 3’168’328 to 3’202’287 of the NGR234 chromosome with the rRNA3 operon, Km^R	Perret et al. 1991

Table S2. Primers for PCR amplifications and sequencing of amplicons.

Restriction sites for cloning are in bold with mismatches to target sequence shown as lowercase.

Primer names	Primer sequences (5' to 3')	Site	Descriptions
**For constructing rRNA mutants**			
C1G-For	CGC**ACtAGt**CCGACGTCACGTT	*Spe*I	To amplify NGR_c34200 at 5’-end of the rRNA1 operon.
C1G-Rev	CTA**GgaTCC**GGCGTATCTTAGAG	*Bam*HI	
C1D-For	ACC**ggATCC**AAAAACAACTGGCCC	*Bam*HI	To amplify NGR_c34280 to *phbC1* at 3’-end of the rRNA1 operon.
C1D-Rev	TAT**CTGCAG**CGCCTCTATCTC	*Pst*I	
C1G-For3	GTTGAAATAGACCGTCTGGC		To confirm rRNA1 deletion. C1G-For3 downstream of NGR_c34200, C1G-Rev3 internal to rRNA1
C1G-Rev3	ACTCAGACACGTCAACAACC		
C1D-For2	GACAAACGAGACAAATCCGC		To confirm rRNA1 deletion. C1D-Rev2 binds in *phbC1*, C1D-For2 binds upstream of $tRNA^{Met}$
C1D-Rev2	TTTCTCGACAGCCTGATGTG		
C3G-For	ATC**aCTAGT**GCCGCCTTCGTAT	*Spe*I	To amplify NGR_c30300 at 3’-end of the rRNA3 operon.
C3G-Rev	CCTG**GGATCC**GTTCCACAGCGA	*Bam*HI	
C3D-For	CTT**GGAtCc**TGTTGCCCGTATG	*Bam*HI	To amplify NGR_c30370 at the 5’-end of the rRNA3 operon.
C3D-Rev	GAA**CtCGAG**TTTGGACGCTGC	*Xho*I	
C3G-For2	GAGAGATAGAGCATGTTGCCC		To confirm rRNA3 deletion. C3G-For2 is upstream of NGR_c30300, C3G-Rev2 binds inside rRNA3
C3G-Rev2	ATCCCGGCGATCCACAAAAGC		
C3D-For2	CTGCCTACCCAAAGAGAGAGG		To confirm rRNA3 deletion. C3D-Rev2 is upstream of NGR_c30370. C3D-For2 binds inside rRNA3
C3D-Rev2	GAAGTTCCATCACACGAGCCC		
Omega	TGATCCGGTGGATGACCTTTTG		Internal to and outwards of the Omega cassettes
**For amplification and sequencing of 16S rRNA genes, promoter and terminator regions**			
rRNA_Pr-Rev	AGTGTTAGTCTCTTGTCAAAACG		To amplify the promoters of rRNA operons
rRNA_Ter-For	TTGCCGACCTGGTGGTTCTG		To amplify the terminators of rRNA operons
rRNA1_Pr-For	GTATCCAGTACCAGCATTTCCG		To target the rRNA1 promoter
rRNA2_Pr-For	TTGCTCTTGCCGTATTCTTAGC		To target the rRNA2 promoter
rRNA3_Pr-For	CGCTGTCGAGTTACGTCTCC		To target the rRNA3 promoter
rRNA1_Ter-Rev	GATGGAGAAGATCAGGAAGGG		To target the rRNA1 terminator
rRNA2_Ter-Rev	CGTAGGGTTCGGAGACTTCG		To target the rRNA2 terminator
rRNA3_Ter-Rev	GTGTTTTCGAGCCCTTCTACC		To target the rRNA3 terminator
16S-For3	AGAGTT**GGATCC**TGGCTCAG	*Bam*HI	To amplify and sequence 16S rRNA genes (Fossou et al. 2016)
16S-Rev3	AAAGGAGG**GGATCC**AGCCG	*Bam*HI	

Cited References

Figurski, D.H., and Helinski, D.R. (1979). Replication of an origin-containing derivative of plasmid RK2 dependent on a plasmid function provided in trans. *Proc. Natl. Acad. Sci. USA* 76**,** 1648-1652.

Fossou, R.K., Ziegler, D., Zeze, A., Barja, F., and Perret, X. (2016). Two major clades of bradyrhizobia dominate symbiotic interactions with pigeonpea in fields of Côte d'Ivoire. *Front. Microbiol.* 7**,** 1793. doi: 10.3389/fmicb.2016.01793.

Perret, X., Broughton, W.J., and Brenner, S. (1991). Canonical ordered cosmid library of the symbiotic plasmid of *Rhizobium* species NGR234. *Proc. Natl. Acad. Sci. USA* 88**,** 1923-1927.

Prentki, P., and Krisch, H.M. (1984). In vitro insertional mutagenesis with a selectable DNA fragment. *Gene* 29**,** 303-313.

Quandt, J., and Hynes, M.F. (1993). Versatile suicide vectors which allow direct selection for gene replacement in Gram-negative bacteria. *Gene* 127**,** 15-21.

Stanley, J., Dowling, D.N., and Broughton, W.J. (1988). Cloning of *hemA* from *Rhizobium* sp. NGR234 and symbiotic phenotype of a gene-directed mutant in diverse legume genera. *Mol. Gen. Genet.* 215**,** 32-37.

Figure S1. Alignment of the rRNA1, rRNA2 and rRNA3 promoters of *S. fredii* strain NGR234

```
          1                            30                            60                            90
          |.........|.........|.........|.........|.........|.........|.........|.........|.........|
                                             UP element          -35                       -10       +1
3,620,478 ttaaaatcagcaacttgtcgttttttgaaGTTTTTTTGAAGAAGGTtgTTGACGtgtctgagttggtgggtcTATAAGcccgatcActga rRNA1
2,758,484 -gtttccgg--tttg-cagaaaac-gtc-T------CC--A-T-CC-------------------------------------------- rRNA2
3,189,571 ga---------tgg--acaaaa--c-atc-------CC--A---CC---------c--ga--ggt-c----------------------- rRNA3

                                                                                            Box B
3,620,568 cgagggcggcggcgctgctggcgacgacgactttcgctctagagtttccgagaga_ttggcggatgctgatttGGGGCTGGGACgcgaGT rRNA1
2,758,394 -------------------------------------------------------_---------------------------------- rRNA2
3,189,481 --------------------------g----------------------------a---------------------------------- rRNA3

                                                     Box A                      Processing stalk
3,620,657 TTTGGCTTTgttgggaacgttgacggggattgtttcctgtcGGTTATTTGACAattgaagattgAGAAAGAGAAACGTGGGCGGCGGAGC rRNA1
2,758,305 ------------------------------------------------------------------------------------------ rRNA2
3,189,391 -------------------------------------c---------------------------------------------------- rRNA3

3,620,747 TCGcgggacctgagagattgggttctggaaagagactttggcggtcacgttttgacaagagactaacacttttatgtttggctcggatac rRNA1
2,758,215 ------------------------------------------------------------------------------------------ rRNA2
3,189,301 ------------------------------------------------------------------------------------------ rRNA3

3,620,837 ctttcgccagccctttgggctgacgggacccgagcctcgcgatacggtatccgtatggcggtttcggcacccttgcccgtcgggcaagaa rRNA1
2,758,125 ------------------------------------------------------------------------------------------ rRNA2
3,189,211 ------------------------------------------------------------------------------------------ rRNA3

3,620,927 aggtttgccgaaacgaacataggtgtgagttctcgtcgattcagacgtgacgtaatgccaatgattgaattctcAACTTGAGAGTTTGAT rRNA1
2,758,035 --------------------------------------------------------------------------AACTTGAGAGTTTGAT rRNA2
3,189,121 --------------------------------------------------------------------------AACTTGAGAGTTTGAT rRNA3
```

Supplementary Figure 1. 540 bp long alignment of the rRNA1, rRNA2 and rRNA3 promoters with the predicted features that are described in the main text shown as grey shaded nucleotide positions. Inverted repeats in conserved Box B are underlined. rRNA1 promoter sequence was arbitrarily selected as consensus, with identical positions shown as hyphens, mismatches and unique gap (underscore) coloured in red. 5'-end of mature 16S rRNA is shown in green uppercase charaters. Left, corresponding nucleotide positions in the NGR234 chromosome sequence archived under the NC_012587 accession number.

Figure S2. Alignment of the 5S rRNA to tRNA^{Met} intergenic regions of the rRNA operons of *S. fredii* strain NGR234

```
           1                            30                            60                            90
           |.........|.........|.........|.........|.........|.........|.........|.........|.........|
           5S rRNA                                                                       ------>
3,626,681  TGCCAGGTCTgcaaagcgcaagaaaaaacactcataagatcatcttctcgacacacgctcggcccaagccgaacaccgggccgctgacca  rRNA1
2,752,281  TGCCAGGTCTgcaaagcgcaagaaaaaacactcataagatcatcttctcgacacacgctcggcccaagccgaacaccgggccgctgacca  rRNA2
3,183,367  TGCCAGGTCTgcaaagcgcaagaaaaaacactcataagatcatcttctcgacacacgctcggcccaagccgaacaccgggccgc_aatac  rRNA3
                                                                                        ------>

           <------                                  -------->   <--------            ---  ------->  <
3,626,771  agcggccttttgcttggccggaccgcaaacagaaacaaacgagattttgctgcgcaaaactcggaccggacaaacgagacaaatccgcac  rRNA1
2,752,191  agcggccttttgcttggccggaccgcaaacagaaacaaacgagattttgctgcgcaaaactcggaccggacaaacgagacaaatccgcac  rRNA2
3,183,278  gcggcccttctgcgtagataagatcagaatagaca_______________________________________________________  rRNA3
           <------

           ------- ---       >--  ------->    <------- --<                        >---- -- - --->   <
3,626,861  ggatttgctcgcacgatacgagacaaattgccaaggcaatttgctcgggtaacgcgggctggagcagccccgccggaccacaggccaaag  rRNA1
2,752,101  ggatttgctcgcacgatacgagacaaattgccaaggcaatttgctcgggtaacgcgggctggagcagccccgccggaccacaggccaaag  rRNA2
3,183,243  ____gtgccttccttacacgagacaaattgc_ttcgcaatttgctcggcacagaccgcaaatccaaaggatttgcgttaccgcg__ataa  rRNA3
                             >--  ------->    <------- --<        >------->  <--------            >--

           ---- -- ----<            >----->    <-----<              tRNA^Met
3,626,951  gccgaggaccggcatacaaggaaccaccgggctgccgcccggttcaagaggtaaCGCGGGGTGG                            rRNA1
2,752,011  gccgaggaccggcatacaaggaaccaccgggctgccgcccggttcaagaggtaaCGCGGGGTGG                            rRNA2
3,183,160  accgcaaagcggtttacgcagaacggataaaccgcaagcggtttacctagatggCGCGGGGTGG                            rRNA3
           ---->   <------<           >------>  <------<
```

Supplementary Figure 2. Alignment of the 5S rRNA (in green) to tRNA^{Met} (in blue) intergenic sequences of NGR234, highlighting the difference between the rRNA3 copy and the corresponding rRNA1 and rRNA2 sequences. When compared to the identical rRNA1 and rRNA2 copies, the 4 gaps (underscores) and 79 mismatches of the rRNA3 intergenic sequence introduced for best alignment are shown in red. Facing arrows placed immediately above and below DNA sequences delimit inverted repeats which sequences are shaded in grey. Left, corresponding nucleotide positions in the NGR234 chromosome sequence archived under the NC_012587 accession number.

Figure S3. Verifying the sequences of the three 16S rRNA genes of NGR234

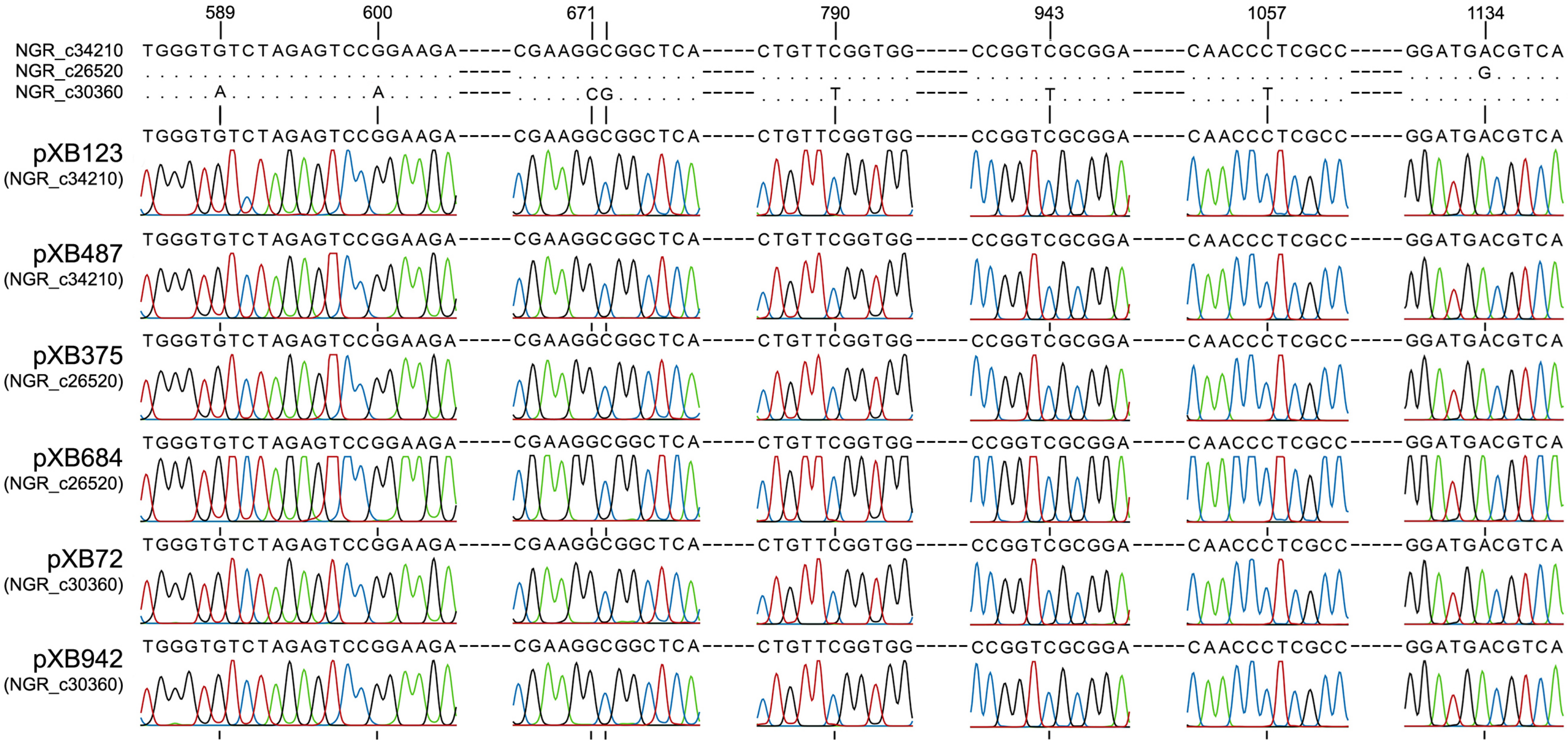

Supplementary Figure 3. To verify the 8 polymorphic positions reported in the NC_012587 sequence (Schmeisser et al. 2009), 16S rRNA genes were amplified and sequenced using overlapping clones of the ordered cosmid library of NGR234 (Perret et al. 1991): NGR_c34210 (rRNA1 locus) was covered by cosmids pXB123 and pXB487, NGR_c26520 (rRNA2 operon) was found in pXB375 and pXB684, while NGR_c30360 of the rRNA3 locus was covered by pXB72 and pXB942. Polymorphic positions in the 16S rDNA genes are shown above the sequence alignment with matching chromatograms obtained with cosmid templates displayed immediately below. Cosmid sequencing confirmed that initial polymorphisms reported in NC_012587 accession were sequencing errors.

Figure S4. Following the development of NGR234 and rRNA deletion mutants on TYA plates

NGR234 **NGRΔrRNA1** **NGRΔrRNA3** **NGRΔrRNA1,3**

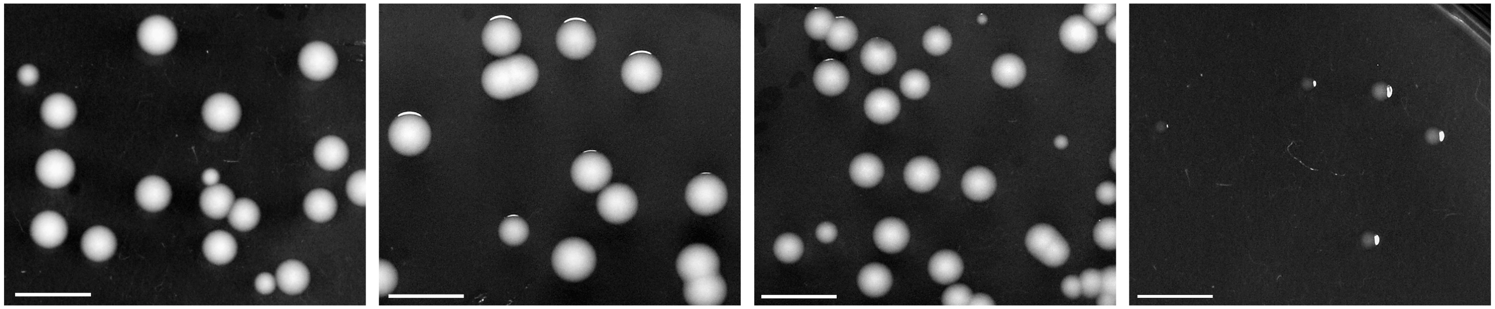

Supplementary Figure 4. Growth of NGR234 and rRNA deletion mutants was monitored on solid media after several days of incubation at 27°C. As explained in the main text, after 5 days of growth on TYA the average colony size was estimated using 30 isolated colonies found on three serial dilutions. Photographs shown above are sections of petris incubated together for 5 days and inoculated with ca. 100 cells. White scale bars correspond to 5 mm. Another experiment using minimal RMS agar medium instead of rich TYA and 7 days incubation, also showed growth of NGRΔrRNA1,3 was impaired when compared to NGR234, although to a lesser extent than when cells were plated on TYA.

Figure S5. Kinetics of nodulation of the NGR234 and rRNA deletion mutants on *Vigna unguiculata*

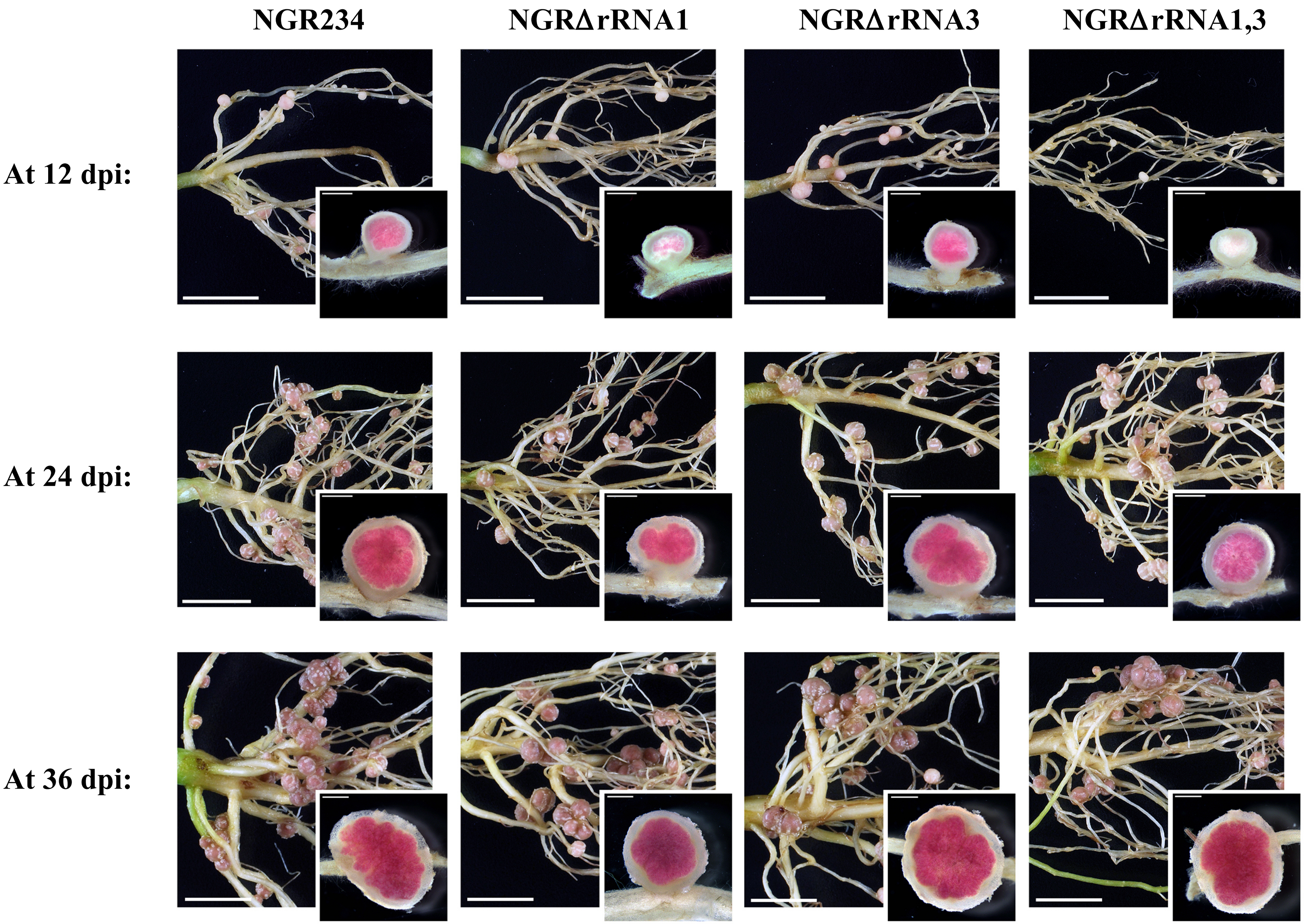

Supplementary Figure 5. To follow nodulation of the parent and rRNA deletion mutants on *V. unguiculata*, plants were harvested at 12, 24 and 36 days post-inoculation (dpi). For each inoculum and time point, at least 12 plants were harvested and data collected on nodule number, nodule fresh weight and shoot dry weight was used to prepare Figure 3 of main text. At each time point, and for each inoculum, root systems of two plants were photographed one of which was selected to prepare Fig. S5. Nodules were imaged using a Leica MZ16 binocular equipped with a Infinity 2 camera. The sections of roots and nodules shown above are organized by inoculum and day of harvest, with white scale bars representing 1 cm (root systems) or 1 mm (nodule sections).
